# Differential Roles for Octanoylated and Decanoylated Ghrelins in Regulating Appetite and Metabolism

**DOI:** 10.1155/2010/275804

**Published:** 2010-03-17

**Authors:** Sara E. Schwandt, Sarath C. Peddu, Larry G. Riley

**Affiliations:** Department of Biology, California State University—Fresno, 2555 E. San Ramon Avenue, Fresno, CA 93720, USA

## Abstract

Since its identification in 1999, ghrelin has been identified in all vertebrate groups. The “active core” of ghrelin is highly conserved among vertebrates, suggesting its biological activity to be also conserved. In fish, both acylated forms of ghrelin have been identified; however, the ratio of the ghrelin-C8 to ghrelin-C10 is not as great as observed in mammals. In the tilapia (*Oreochromis mossambicus*), ghrelin-C10 is the major form of ghrelin. Since fish are known to inhabit every ecological niche on earth, studies on fish have provided valuable insight into vertebrate physiology in general; it is likely that understanding the role of both acylated forms of ghrelin, in more detail, in fish will result into novel insights in the biology of ghrelin within vertebrates. In this paper we discuss ghrelin's role in regulating appetite and metabolism in fish, in general, and provide evidence that the two tilapia ghrelins exhibit different biological roles.

## 1. Introduction

The discovery of ghrelin in 1999 [1] broadened our understanding of energy metabolism in vertebrates, resulting in a shift in our approach to investigat the regulation of energy homeostasis in vertebrates. In mammals, two major forms of ghrelin are found in circulation: octanoylated ghrelin at Ser-3 and des-acyl ghrelin [2]. The acyl modification is essential for biological activity [1]; however, some findings provide evidence that des-acyl ghrelin exhibits some biological action [3–7]. Ghrelin has also been identified in all vertebrate classes including sharks [8]. As seen in mammals, the ghrelins identified in other vertebrates are uniquely acylated by either octanoic or decanoic acid on the third amino acid residue from the N-terminus. Indeed, the first seven amino acids of N-terminal region—“active core”—in all vertebrate ghrelins display high sequence homology [8], suggesting that the biological actions of ghrelin are highly conserved across vertebrates. Interestingly, fish ghrelins possess an amide structure on the C-terminus which is not found in tetrapod and shark ghrelins [8]. In the Mozambique tilapia (*Oreochromis mossambicus*), a warm water teleost (fish), we have identified two forms of ghrelin, with identical amino acid sequences, acylated by octanoic or decanoic acid, ti-ghrelin-C8 and ti-ghrelin-C10, respectively [9]. It appears that ti-ghrelin-C10 is the primary form of ghrelin in tilapia. A recent report in goldfish identified 11 different forms of ghrelins; a 17-residue octanoylated form being the predominate form [10]. This finding in goldfish is similar to other vertebrates, with ghrelin-C8 being the major form of ghrelin. In humans, 25% of the ghrelin isolated from the stomach is ghrelin-C10 [11], in the bullfrog, ghrelin-C10 represents 33% of the total ghrelin [12], and in the Japanese eel, ghrelin-C10 represents 44% of the total ghrelin [13]. Due to the evolutionary diversity and breadth of ecological niches occupied by fish; studies using fish as a model have been a rich source of information on the mechanisms that regulate vertebrate growth, metabolism, and development [14–17]. Furthermore, the fact that the ghrelin gene and peptide exhibit high structural similarities and biological actions across vertebrates, suggest that ghrelin is an evolutionary conserved, essential hormone in vertebrates. However, our understanding of ghrelin's basal biological role in vertebrates is unclear. Therefore, studies on fish will provide an evolutionary role for ghrelin and provide insight into the basal function of ghrelin within all vertebrates. This paper will highlight our current understanding of ghrelin's role in food intake and metabolism in fish and provide evidence on how the decanoylated ghrelin plays a significant role in regulating overall energy homeostasis. The reader is directed to reviews focusing on ghrelin sequence identity and other biological actions in nonmammalian vertebrates [8, 18]. 

In mammals, ghrelin has been shown to exhibit a range of actions on cardiovascular, gastrointestinal, and pancreatic functions, as well as lipogenic and glucogenic actions [19]. In mammals, it is suggested that the main physiological function of ghrelin is to stimulate growth hormone release from the pituitary and increase food intake [20]. However, some reports demonstrate that ghrelin does not play a primary role in initiating feeding nor as a regulator of feeding patterns [21]. Indeed, accumulating data suggests that ghrelin's role may be directed to maintain overall energy homeostasis as observed in humans [22, 23] and in pigs [24].

Ghrelin's first reported action was as a potent growth hormone (GH) secretagogue [1]. Since then, similar findings have been reported in fish. We first reported in fish that rat ghrelin-C8 stimulated the release of GH from cultured tilapia pituitaries after 8 h of incubation [25]. Both eel and tilapia ghrelin-C8 stimulated the release of GH from static tilapia pituitary cultures after 2 h of incubation [9, 13]. Recently, we demonstrated that ti-ghrelin-C10 appears to be more effective than ti-ghrelin-C8 in elevating plasma GH levels and in stimulating GH release from tilapia pituitaries [26]. However, these responses occur 4-5 h after treatment. Unlike the delayed response observed in tilapia, intraperitoneal (i.p.) injections of homologous ghrelin-C8 in goldfish and rainbow trout significantly elevated plasma GH levels within 30 min [18, 27]. At least in fish, only the tilapia pituitary releases prolactin (PRL) after ghrelin treatment. Both eel and tilapia ghrelins stimulated the release of PRL from cultured tilapia pituitaries [9, 13, 25]. Similar findings were observed in the bullfrog [12], but not in dispersed rat pituitary cells [1]. These findings clearly suggest that the response to ghrelin is species specific, but what needs to be more clearly investigated in fish is; does ghrelin exhibit the same stimulatory effect on GH release during altered physiological states (i.e., fasting or stress). We have recently reported for the first time using the hybrid striped bass model that ghrelin was equally effective in stimulating GH release from pituitaries of fed and starved animals. Furthermore, both plasma levels of ghrelin and GH were significantly elevated in fasted cold-banked animals [28]. Suggesting that ghrelin is driving the elevation of plasma GH levels during fasting as proposed in mammals [29] or regulating energy partitioning during catabolic states [28]. 

**Figure 1:**
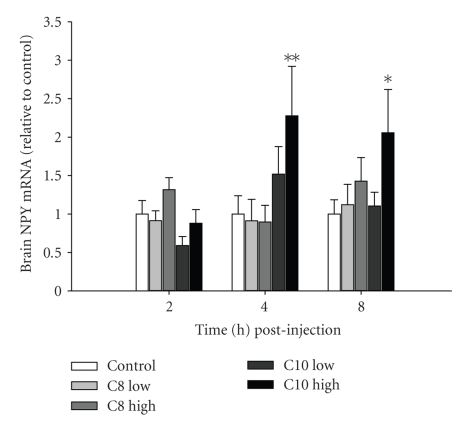
The effect of tilapia ghrelin-C8 and ghrelin-C10 on brain NPY mRNA levels. Two different doses of both ghrelins, low (1 ng/gm) and high (10 ng/gm), were administered by a single i.p. injection and samples were collected a 2, 4, and 8 h postinjection. mRNA levels were normalized to the house keeping gene, acidic ribosomal phosphoprotein P0 (ARP). Vertical bars represent mean ± SEM (*n* = 8–10). ∗, ∗∗ are significantly different from time-matched control at *P* < .05 and <.01, respectively (2-way ANOVA).

As mentioned above, several reports in mammals have demonstrated that acute ghrelin treatment stimulates food intake [20]. However, in teleosts, ghrelin's orexigenic actions have not been well studied and appear not to be widespread. The only report of ghrelin exhibiting rapid orexigenic actions—as seen in mammals—is in goldfish [30, 31], whose actions have been shown to be mediated by neuropeptide Y (NPY) [32]. In tilapia, we have been unable to observe an acute increase in food intake following ti-ghrelin-C8 or ti-ghrelin-C10 treatment (unpublished observations). However, we have observed in tilapia given a single i.p. injection of ti-ghrelin-C10 (10 ng/gm BW) a significant increase in brain NPY mRNA levels 4 and 8 h (*P* < .01 and *P* < .05, resp.) postinjection was observed, whereas ti-ghrelin-C8 did not alter NPY mRNA levels (Figure 1). The inability of ti-ghrelin to stimulate acute food intake may likely be a result from the site of treatment. Centrally administered ghrelin is very potent in stimulating food intake in mammals [30, 33] and goldfish [10, 30], whereas peripherally injection of ghrelin is less effective in stimulating food intake [33]. It may be likely that orexin, which has not yet been identified in tilapia, is mediating acute food intake in tilapia. In goldfish, it has been shown that ghrelin and orexin interact to stimulate feeding [34]. It is of interest to identify orexin in tilapia and investigate its action on food intake. We have shown previously that 21 days of ti-ghrelin-C10 (ti-ghrelin-C8 had no effect) treatment significantly increased food intake and adiposity in liver and muscle tissue in tilapia [35]. Similar findings have been observed in rodent models [33]. In addition, 21 days of ti-ghrelin-C10 treatment did not alter plasma GH levels, but plasma levels of insulin-like growth factor-I (IGF-I) were significantly reduced, suggesting that ti-ghrelin-C10 is inhibiting growth in favor of storing metabolic energy as fat; generating a positive energy balance. Interestingly, ti-ghrelin-C8 treatment significantly increased pituitary GH mRNA levels [35]. In rats receiving a continuous i.c.v. infusion of ghrelin for 12 days, plasma GH levels were not altered [36]. Furthermore, in rainbow trout, a single i.p. injection of ghrelin failed to stimulate appetite, however, plasma ghrelin levels were positively correlated with growth rate and negatively correlated with plasma GH and IGF-I, suggesting that ghrelin may be linked to growth and metabolism and not appetite [37]. It would be of interest to see if the two acylated forms of ghrelin identified in other vertebrates exhibit different biological effects as we have observed in tilapia. What is interesting in tilapia is that we have shown that the GHS-R antagonist, [D-Lys^3^]-GHRP-6, completely abolished the stimulation of GH release by both forms of tilapia ghrelin from dispersed pituitary cells [26]. These findings suggest that there is likely a second ghrelin receptor exhibiting higher specificity for ti-ghrelin-C10 with a different tissue distribution pattern than the one currently identified (GRLN-R). In the rat, ghrelin and des-octanoyl ghrelin but not a GHS-R1a agonists (L-163-255) induced adipogenesis independent of GH secretion, suggesting that ghrelin's adipogenic action is likely mediated by a novel receptor that is distinct from GHS-R1a [3]. 

**Figure 2:**
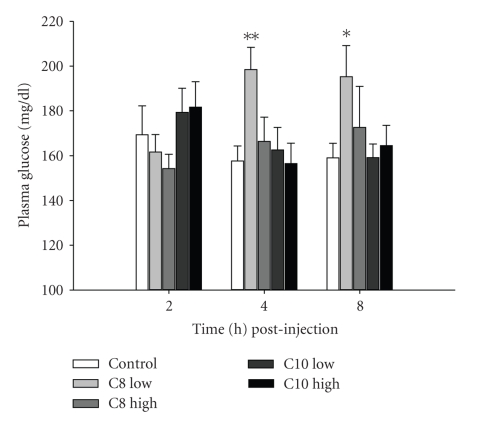
The effect of tilapia ghrelin-C8 and ghrelin-C10 on plasma glucose levels. Two different doses of both ghrelins, low (1 ng/gm) and high (10 ng/gm), were administered by i.p. injection and samples were collected a 2, 4, and 8 h postinjection. Vertical bars represent mean ± SEM (*n* = 8–10). ∗, ∗∗ are significantly different from time-matched control at *P* < .05 and <.01, respectively (2-way ANOVA).

**Figure 3:**
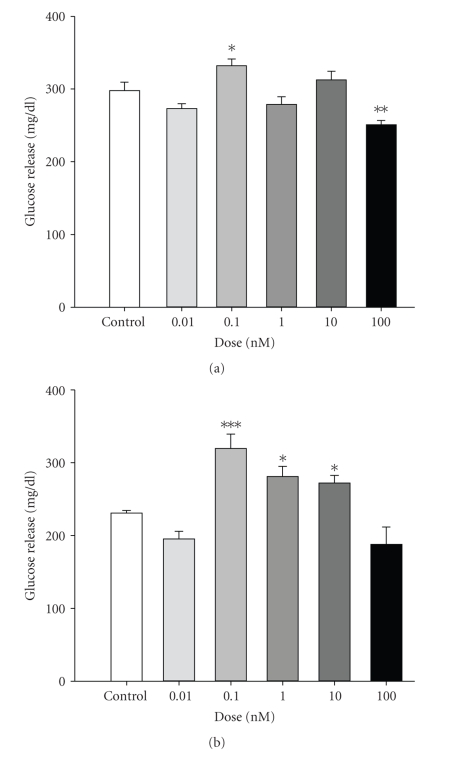
The effect of tilapia ghrelin-C8 (a) and ghrelin-C10 (b) on glucose release from cultured tilapia hepatocytes. Hepatocytes were exposed to ti-ghrelins for 6 h at that time culture media was collected and analyzed for glucose content. Vertical bars represent mean ± SEM. ∗, ∗∗ are significantly different from control at *P* < .05 and <.01, respectively (1-way ANOVA). *n* = 8–10.

**Figure 4:**
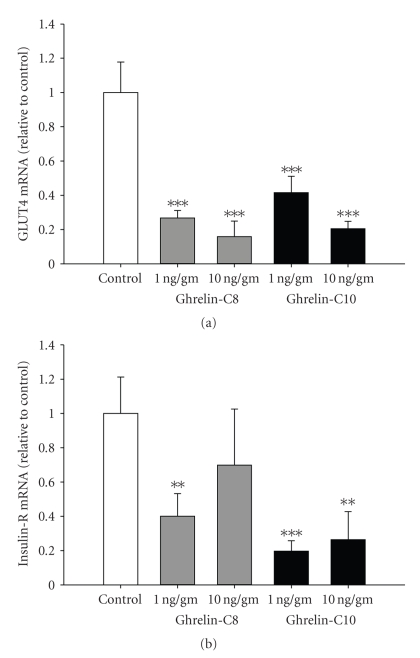
Effect of ghrelin-C8 and ghrelin-C10 on muscle GLUT4 (a) and insulin-R (b) mRNA levels 2 h post-injection. mRNA levels were normalized to the house keeping gene ARP. Vertical bars represent mean ± SEM (*n* = 5-6). ∗, ∗∗, ∗∗∗ significantly different from control at *P* < .05, .01, and .001, respectively (1-way ANOVA).

In mammals, ghrelin has been shown to play a role in glucose homeostasis by exhibiting diabetogenic actions, by inducing hyperglycemia in humans [38]. We have observed similar effects in tilapia. In tilapia given a single i.p. injection of ti-ghrelin-C8 (1 ng/gm BW) plasma glucose levels were significantly elevated at 4 and 8 h (*P* < .01 and *P* < .05, resp.) postinjection; the high dose (10 ng/mL) was without effect (Figure 2). It is possible that the most effective dose of ti-ghrelin-C8 on plasma glucose levels is in the picogram range. It would be of interest to determine the dose response range of ti-ghrelin-C8 on plasma glucose levels. In cultured tilapia hepatocytes, ti-ghrelin-C8 (0.1 nM) significantly (*P* < .05) stimulated glucose release, whereas at 100 nM ti-ghrelin-C8 significantly (*P* < .05) reduced the release of glucose after a 6 h incubation (Figure 3(a)). Interestingly, ti-ghrelin-C10 (0.1–10 nM) significantly stimulated the release of glucose from cultured tilapia hepatocytes (Figure 3(b)), but without altering plasma glucose levels (Figure 2). This is the only report in any teleost describing the effect of ghrelin on glucose metabolism. Future work is needed to elucidate ghrelin's role in glucose metabolism in fish, especially since fish are considered to be glucose intolerant [39], and therefore fish could be an ideal alternative model for diabetes research. We have been able to detect both GHS-R1a and GHS-R1b transcripts in tilapia liver [26]. In both porcine and rat hepatocytes, ghrelin stimulated the release of glucose [4]. However, a potent GHS, hexarelin, failed to alter plasma glucose levels. Gnanapavan and colleagues failed to identify the GHS-R1a in human liver, whereas the GHS-R1b was highly expressed; suggesting that the hyperglycemic action of ghrelin in mammals may be mediated through GHS-R1b or an unknown orphan GHSR [40]. Therefore, it is likely that the observed differential response of tilapia hepatocytes to the tilapia ghrelins is that an unknown orphan GHSR is present in tilapia that exhibits higher affinity to one ghrelin over the other. In spite of the differential effects of the tilapia ghrelin's on plasma glucose levels, we have observed that both ti-ghrelin-C8 and ti-ghrelin-C10 significantly reduced muscle mRNA levels of a putative tilapia glucose transport protein (GLUT4) and the insulin receptor (Figure 4). To our knowledge this is the first report in any vertebrate. Whether protein levels of GLUT4 and insulin receptor are changed following ghrelin treatment needs to be investigated. 

The existence of ghrelin, GHS-R1a and GHS-R1b in fish suggests that the actions of ghrelin and GHSRs are conserved across vertebrate species and likely exhibit fundamental biological functions within vertebrates [41, 42]. Our data show that the two forms of tilapia ghrelin (octanoylated and decanoylated) exhibit different biological actions but that they may function together to maintain overall energy homeostasis in tilapia. It is of interest to investigate if the decanoylated form of ghrelin found in other vertebrates exhibits different biological activity than the octanoylated form. Furthermore, how different physiological states within the animal alter the circulating levels of these two different tilapia ghrelin's needs to be investigated. Currently, however, we are unable to differentiate between circulating ti-ghrelin-C8 and -C10 levels in our radioimmunoassay.
